# Contemporary Management of Locally Advanced and Recurrent Rectal Cancer: Views from the PelvEx Collaborative

**DOI:** 10.3390/cancers14051161

**Published:** 2022-02-24

**Authors:** PelvEx Collaborative

**Keywords:** rectal cancer, locally advanced rectal cancer, recurrent rectal cancer, diagnostics, surgical management, surgical outcomes, perioperative care, quality of life

## Abstract

**Simple Summary:**

Pelvic exenteration is a complex procedure performed for the management of advanced pelvic cancers. It often involves the resection of several pelvic organs and can be associated with a high morbidity and impact on the patient’s quality of life. The development of better diagnostics, improved chemotherapy and radiotherapy regimens, combined with advanced surgical strategies have improved surgical and survival outcomes. This article highlights current and future management options.

**Abstract:**

Pelvic exenteration is a complex operation performed for locally advanced and recurrent pelvic cancers. The goal of surgery is to achieve clear margins, therefore identifying adjacent or involved organs, bone, muscle, nerves and/or vascular structures that may need resection. While these extensive resections are potentially curative, they can be associated with substantial morbidity. Recently, there has been a move to centralize care to specialized units, as this facilitates better multidisciplinary care input. Advancements in pelvic oncology and surgical innovation have redefined the boundaries of pelvic exenterative surgery. Combined with improved neoadjuvant therapies, advances in diagnostics, and better reconstructive techniques have provided quicker recovery and better quality of life outcomes, with improved survival This article provides highlights of the current management of advanced pelvic cancers in terms of surgical strategy and potential future developments.

## 1. Introduction

Pelvic exenteration is a complex operation performed for locally advanced and recurrent pelvic cancers [1]. The procedure involves an the en bloc resection of at least two pelvic organs with subsequent reconstruction and/or diversion of bowel/urinary/sexual functions. The goal of surgery is to achieve clear margins; therefore, adjacent or involved organs, bone, muscle, nerves and vascular structures may be resected [2,3]. While these extensive resections are potentially curative, they are associated with significant morbidity [4]. A multidisciplinary team approach is essential to optimise patients pre-operatively and during their recovery process [5]. More recently, there has been a move to centralize care to specialized units, as this facilitates a better integration of prehabilitation protocols, subspecialty involvement and a greater emphasis on research and quality of life assessments [5,6,7,8].

The COVID-19 pandemic has disrupted patient access to healthcare worldwide, despite efforts to maintain essential cancer care during this time [9]. Early studies have shown a concerning delay in cancer diagnosis and treatment during the initial ‘waves’ of the pandemic [10,11]. This is predicted to cause an increase in the proportion of patients with more advanced stages of cancer [9]. This may challenge our already strained healthcare services, with protected surgical beds and the availability of intensive care facilities essential for maintaining complex surgical care [12].

Advancements in pelvic oncology and surgical innovation have redefined the boundaries of pelvic operations [13]. Aggressive surgical techniques including extended pelvic exenteration (bony/vascular resection) and cytoreductive surgery with or without hyperthermic intraperitoneal chemotherapy (CRS-HIPEC) reported improved five-year overall survival rates [14,15,16,17,18]. Improved neoadjuvant therapies and advances in imaging techniques, navigational technology and artificial intelligence facilitate the increased downstaging of advanced neoplasms, more judicious patient selection and/or greater surgical precision [19,20]. Reconstructive techniques provided quicker recovery and better quality of life outcomes [21]. This article aims to provide insight into the contemporary management of advanced pelvic cancers in terms of surgical strategy and future developments.

## 2. Contemporary Management Strategies

### 2.1. Pre-Operative Strategies

#### 2.1.1. Tumour Staging and Predicting Resectability

Precise tumour staging is essential to determine prognosis and enables a more focused assessment of the available management options [13]. Local staging for rectal cancer is most accurately established with magnetic resonance imaging (MRI), with a sensitivity and specificity of up to 100% and 98%, respectively, for T3 and T4 disease. Staging for distant metastases is routinely performed with computerised tomography (CT) of the thorax, abdomen and pelvis [14]. Recently, the concept of whole-body MRI (WB-MRI) has been postulated, as a radiation-free alternative for surveying cancer patients. Furthermore, WB-MRI is shown to be highly accurate in the detection of bone metastases. Despite this, WB-MRI has not been widely utilized [15,22]. Ongoing studies are comparing it to positron emission tomography (PET-CT), especially in the setting of mucinous adenocarcinoma, peritoneal malignancy and other PET-insensitive neoplasms [15,23].

The use of MRI of the chest/thorax is not widely available and is subject to significant operator variation [16]. However, the role of MRI of the abdomen/pelvis is well-established [24]. MRI liver is used to characterise equivocal lesions on CT [25]. Therefore, the role of WB-MRI may evolve over time as a viable alternative to CT.

Regardless of modality, good communication and feedback between surgical and radiological departments is critical in the assessment of patient operability and for predicting resectability. Expertise is required to devise a radiological roadmap that incorporates surgical planes, highlighting potential issues and ensuring negative margins. A roadmap for R0 excision should be tailored to the maximum possible disease extent previously identified on sequential MRI imaging, regardless of down-staging post-neoadjuvant treatment. The rationale behind this is based on the knowledge that radiologically occult microscopic foci of viable tumour cells can occasionally persist beyond down-staged tumour margins (peri-tumoral scar tissue). For this reason, areas of fibrosis in contact with tumours on post-treatment imaging should be considered to have malignant potential and included in the extended resection [26,27,28,29,30]. The radiological roadmap should be tailored individually to each patient, accounting for their anatomy, tumour extent and co-morbidities. ‘BONVUE’ is a helpful acronym used to ensure the radiology team includes a description of bones, organs, nerves, vessels, ureters and extra (tumour sites) in the development of a roadmap [31].

#### 2.1.2. Neoadjuvant Therapy

Total neoadjuvant therapy (TNT) is increasingly utilized for the downstaging of locally advanced rectal cancer (LARC) [32]. Recent studies highlight that TNT may improve outcomes by increasing patient compliance to therapy, reducing tumour stage, and exposing patients to earlier chemotherapy [19]. TNT strategies vary between centres, with some advocating chemo-induction prior to long-course chemoradiotherapy (induction), while others favour consolidation chemotherapy (long-course chemoradiotherapy followed by chemotherapy) [33]. Two recent meta-analyses demonstrated an improved pathological complete response (pCR) in patients who underwent TNT compared to conventional treatment [19,32]. Recently, the PelvEx Collaborative has become involved in two randomised controlled trials examining the role of TNT in the setting of recurrent rectal cancer. PelvEx II trial (NCT 04389088) and GRECCAR 15 have started to recruit patients, and their outcomes will influence the future management of patients with recurrent rectal cancer. The PelvEx II trial is a multi-centre, open-label, randomised, controlled, parallel arms clinical trial of induction chemotherapy followed by chemoradiotherapy versus chemoradiotherapy alone as neoadjuvant treatments for locally recurrent rectal cancer (LRRC). Similarly, the GRECCAR 15 trial is a phase III randomised trial aiming to evaluate chemotherapy followed by pelvic re-irradiation versus chemotherapy alone as neoadjuvant treatment in LRRC [34].

The treatment response to neoadjuvant chemoradiotherapy is highly variable, with up to 20% of rectal tumours exhibiting a complete resistance [35]. The activation of mutations in genes in the phosphatidylinositol 3-kinase (PI3K) and MAP kinase (MAPK) signalling pathways is shown to modulate treatment response and clinical outcomes in locally advanced rectal cancers [36]. Gene mutations associated with an increased response to TNT include ARID1A, PMS2 and JAK1, whereas those associated with resisting treatment include KDM6A, ABL1 and DNMT3A [35]. In addition, work on the relationship between treatment resistance and the microbiome is emerging. One study found greater deposits of fusobacteria in an RNA analysis of pre-treatment tumours in intermediate and poor responders to neoadjuvant therapies [36]. This opens several promising avenues that can be investigated, especially as genomic sequencing increasingly influences the preferred neoadjuvant regimen or agent.

Programmed cell death-1 (PD-1) is a cell receptor found on the surface of activated T-cells, pro-B cells and macrophages that contains at least two ligands; programmed death-ligand 1 (PD-L1) and programmed death-ligand 2 (PD-L2). The binding of these two ligands to the PD-1 receptor results in T-cell deactivation and subsequent tumour cell evasion, preventing a host attack on its own immune system [37]. PD pathway blockade, in order to prevent immune evasion, is a novel method that can also be considered [37,38]. A recent meta-analysis revealed prolonged survival rates in patients with dMMR/MSI-H mCRC receiving anti-PD-1 inhibitor monotherapy [37]. Despite these promising results, many questions remain. dMMR/MSI-H tumours only account for 5% of mCRC and further research is required to extend the benefit of immunotherapy into a broader, microsatellite stable population [39].

#### 2.1.3. Prehabilitation

Perioperative strategies to optimise the outcomes of patients undergoing pelvic exenteration or extended resection for pelvic cancers is critical to maximising treatment success [40]. Pre-existing co-morbidities are associated with poorer outcomes [25]. Prehabilitation can be defined as the process of ‘optimising physical functionality preoperatively to enable the individual to maintain a normal level of function during and after surgery’. It encompasses a combination of exercise, nutrition and psychosocial interventions [41,42]. A meta-analysis of fifteen studies revealed a significantly lower hospital length of stay in patients undergoing cancer surgery, demonstrating an accelerated post-operative recovery in patients exposed to prehabilitation in the pre-operative period [42]. To optimise patient outcomes, multidisciplinary collaboration is essential, incorporating opinions from members of the anaesthetic and surgical teams, nursing staff and other allied health professionals [40].

Surgical patients with a poor functional capacity, determined by oxygen consumption at anaerobic threshold (AT) during cardiopulmonary exercise testing (CPET), experience poorer post-operative outcomes. The identification of high-risk surgical patients allows for the appropriate planning of their perioperative care, subsequently reducing the risk of mortality or severe complications in the post-operative period [43]. Pre-operative cardiopulmonary exercise testing (PCPET) allows us to assess exercise capacity whilst identifying causes of exercise limitations. Information acquired from PCPET can be invaluable for estimating the risk of perioperative events [44]. Anaerobic threshold (AT) indicates the status of the patient’s aerobic fitness and is predictive of perioperative outcomes [43]. The PelvEx Collaborative previously outlined five consensus recommendations to optimise preoperative assessment and preparation in patients undergoing pelvic exenteration [40]:

- Where possible, the anaesthetist undertaking the case should personally pre-assess the high-risk patient undergoing exenteration;
- When available, CPET should be utilised to assess functional capacity pre-operatively;
- Pelvic exenteration can be undertaken in patients who have demonstrated an adequate CPET result and have been deemed low risk for severe perioperative morbidity;
- Patients with more than two cardiac risk factors and poor functional capacity should undergo imaging stress-testing prior to surgery;
- Formal cardiology assessment is not routinely required in patients undergoing pelvic exenteration.

Cancer-related malnutrition occurs secondary to anorexia, nausea, vomiting, metabolic disorders and psychological factors in patients undergoing major oncological surgery. Two cohort studies identified that 32.5% and 24% of their populations, respectively, were malnourished before exenteration when assessed by the subjective global assessment (SGA) tool [25]. The optimisation of nutritional and metabolic state prior to surgery contributes to improved perioperative outcomes and is being increasingly employed as part of pre-operative MDT disease management [45]. Malnutrition during neoadjuvant therapy was also associated with adverse perioperative outcomes, including reduced tumour response, poor treatment tolerance and increased morbidity [46,47]. Early identification and treatment of malnutrition was shown to improve outcomes, lower infection rates, shorten hospital stay and improve wound healing [48].

### 2.2. Operative Strategies

#### 2.2.1. Pushing the Boundaries of Exenterative Surgery

Negative resection margins (R0) are the single most important prognostic factor in predicting long-term survival in patients undergoing pelvic exenteration [1,5,7,8,13,49,50,51,52,53,54,55]. The goal of exenterative surgery is to resect all involved organs/structures whilst balancing this radicality with an acceptable risk profile and postoperative quality of life. In recent decades, more extensive procedures are being performed, with better patient education and counselling regarding the risks [14,17]. Various surgical techniques have been developed to facilitate en bloc resection of ‘higher and wider’ pelvic tumours beyond traditional mesorectal planes. These include high sacrectomy, pubic bone resection and lateral compartment excision, often involving major neurovascular structures [17]. Low sacrectomy (below S3) is performed routinely by exenterative surgeons, demonstrating relatively low complication rates regardless of patient positioning [13]. Similarly, high sacrectomy is shown to be safe and efficacious without compromising R0 rates and is no longer considered a contraindication to surgery [56]. Several studies showed that an R0 resection can be achieved in 55–80% of patients with recurrent rectal cancer undergoing exenterative surgery, translating to a 5-year overall survival of 28–50% [57,58,59]. Several specialised units have adopted novel techniques for en bloc sacral resection that minimise morbidity by avoiding complete sacrectomy. Proposed methods include anterior sacrectomy (resection of the anterior cortex to preserve nerve roots), segmental sacrectomy or high subcortical sacrectomy (HiSS) [60,61]. In the past, the involvement of the pelvic sidewall was considered an absolute contraindication for surgery due to bony limitations and the presence of major neurovascular structures [54]. However, better reconstructive methods have allowed for these more radical resections [62]. Increasingly, the selective en bloc resection of the pelvic side-wall structures, including the internal iliac vessels, piriformis and obturator internus muscles, ischium, and sacrotuberous/sacropspinous ligaments is being performed [63].

Historically, the presence of hydronephrosis, gross lower limb oedema, and invasion of the sciatic notch or involvement of the aortoiliac axis suggested inoperable disease. Various studies have demonstrated the safety and efficacy of major extra-anatomic resections involving these structures in selective patients [64,65,66]. En bloc sciatic nerve and/or lumbosacral trunk resections for tumours extending laterally into the piriformis muscle have demonstrated similar R0 rates to central pelvic tumours [13]. Functional outcomes are always a concern when undertaking these radical resections; however, almost all patients undergoing complete sciatic nerve resection regain mobility post-operatively following intensive physiotherapy and orthotics input [64]. En bloc major vascular resections of the aortoiliac axis are also shown to be feasible in select patients in specialised centres, with an R0 rate of 81.8% reported in one study [65].

While the extent of resection is theoretically limitless, it is imperative that morbidity is minimized and, therefore, operations are tailored to each patient. As advancements in reconstructive methods and rehabilitative systems are made, the indications and contraindications for surgery are constantly changing [14]. Currently, absolute contraindications to resection include: poor performance status, bilateral sciatic nerve involvement and/or circumferential bone involvement. Relative contraindications include: encasement of external iliac vasculature, high sacral involvement (above S2), extension through the sciatic notch, and/or unresectable extra-pelvic metastases [30].

#### 2.2.2. Intra-Operative Radiation Therapy (IORT)

IORT delivers a single high-fraction dose (10–20 Gy) of radiation directly to anatomical targets deemed as having the potential for high recurrence risk [67]. IORT is usually administrated to patients with either no or a limited volume of metastatic disease [68]. The addition of IORT to conventional multi-modal treatment strategies has been shown to achieve excellent local control outcomes in ‘select’ patient cohorts [67,69]. The utilization of IORT in the setting of gross residual (R2) disease is of limited value.

IORT can be delivered via two methods: high-dose-rate brachytherapy or intraoperative electron beam radiotherapy (IOERT). The evidence for IORT stems from positive prospective long-term data from several international units who administer it when there is concern for threatened margins [70]. A recent systematic review suggested that IORT may improve oncological outcomes in advanced and recurrent colorectal cancers, offering better local control, disease-free survival and overall survival with no associated increase in severe complications [67].

#### 2.2.3. Pelvic Exenteration in the Setting of Peritoneal Disease

Current treatment options for patients with peritoneal metastases include supportive care, palliative systemic chemotherapy, pressurised intraperitoneal aerosol chemotherapy (PIPAC), and cytoreductive surgery (CRS) combined with heated intraperitoneal chemotherapy (CRS-HIPEC). When treated with modern systemic chemotherapy, these patients have a median survival of thirteen months [71]. Patients with low-volume peritoneal disease may be considered for CRS-HIPEC with curative intent, whereas those with more extensive disease may benefit more from PIPAC [72].

CRS-HIPEC is a well-established treatment modality for patients with synchronous or metachronous colorectal peritoneal metastases [73]. While it is associated with a long-term survival benefit, high rates of morbidity were observed to range from 12 to 65% [74]. Traditionally, CRS-HIPEC is not recommended in those needing a pelvic exenteration [75]. Recent studies suggest the feasibility and safety of these two procedures being performed simultaneously, with an acceptable level of morbidity [74,75]. The PRODIGE-7 trial compared CRS and CRS-HIPEC in patients with peritoneal metastases. All patients received at least six months of oxaliplatin-based systemic chemotherapy, with the CRS-HIPEC arm receiving additional HIPEC with oxaliplatin for 30 min. Median survival was 41.7 and 41.2 months with and without HIPEC, respectively [76]. This study demonstrated that oxaliplatin-based HIPEC did not improve survival in this cohort of patients. Tuech et al. recently demonstrated a complete cytoreduction in all patients undergoing CRS-HIPEC and TPE, with R0 margins achieved in 81.2%. Despite these promising results, severe complications occurred in 56.2% of patients and post-operative mortality was 12.5% [74].

Further research is required via a multi-centre approach to determine the optimal candidates for this approach [77]. The PRODIGE 7 trial demonstrated no clear benefit in the use of oxaliplatin-based HIPEC in addition to standard chemotherapy. However, more data are required to evaluate if other HIPEC chemotherapeutic regimens are better.

#### 2.2.4. Oligometastatic Disease

For patients presenting with rectal cancer, 15–20% will have synchronous liver metastases [52]. The optimal management of these patients is subject to debate and often dependent on local resources and expertise [51]. Historically, surgical resection in patients with LARC or LRRC was confined to patients without metastatic disease. However, simultaneous hepatic resection was reported to be technically feasible with an acceptable morbidity and mortality rate when performed on select patients [52,78]. The median cancer-specific survival after liver resection for colorectal cancer with liver metastases was reported as 42.5 months, with disease tending to recur in patients with poor differentiation of the primary tumour, positive lymph nodes and higher amounts of liver metastases [79].

The PelvEx Collaborative observed an R0 resection in 73.5% of pelvic exenterations and 66.4% of liver resections among 128 patients with synchronous liver metastases. The 5-year overall survival for patients in whom an R0 resection was achieved was 54.6% in comparison to 20% for those with an R1/R2 resection. This was the first multi-centre study that demonstrated the safety and feasibility of simultaneous liver resection, with acceptable morbidity and mortality rate [52].

Debate around the optimal treatment of colorectal lung metastases also remains. [80]. The majority of these metastases are suitable for surgical resection, with reasonable 5-year overall survival rates [81]. A recent meta-analysis demonstrated comparable survival rates between both the surgical and non-surgical management of colorectal pulmonary metastases, contrary to earlier evidence suggesting a benefit of resection [80]. The recently published LaIT-SABR study aimed to identify predictive factors of sterotactic ablative radiotherapy (SABR) response in patients with colorectal lung metastases and investigate the rates of progression to polymetastatic disease [82]. Their results support the use of SBRT in this cohort of patients as it was associated with a delay in progression to polymetastatic disease. Further prospective studies are necessary to obtain a better understanding of the long-term effect of lung metastasectomy in metastatic colorectal cancer [83].

#### 2.2.5. Minimally Invasive Surgery (MIS)

Minimally invasive surgical modalities have evolved considerably in recent years, particularly regarding pelvic procedures [84]. A recent meta-analysis published by the PelvEx Collaborative investigated the current evidence regarding the use of MIS techniques such as laparoscopy and robotic surgery in pelvic exenteration. It was concluded that MIS exenteration was associated with reduced intra-operative blood loss and hospital length of stay while having no adverse effect on resectability [1]. Since robotic-assisted pelvic exenteration was first described in 2013, there are increasing numbers of case reports and series demonstrating its safety and feasibility [85]. The current evidence in the literature suggests an acceptable operative time, blood loss and a range of R0 rates [84,86,87,88,89,90,91]. Laparoscopic pelvic exenteration has been associated with reduced blood loss, faster recovery and an acceptable length of stay; on the contrary, in well-selected patients, the learning curve is steep [92]. Robotic-assisted surgery facilitates a more ergonomic and visually enhanced platform [93].

In the PelvEx Collaborative meta-analysis comparing MIS techniques to the open approach, 78.1% underwent open exenteration while 21.8% had an MIS exenteration analysis among 170 patients. MIS exenteration was associated with a longer operating time but substantially less blood loss. MIS exenteration was also associated with a significantly reduced overall morbidity rate (56.7% versus 88.5%) and a short post-operative length of stay (6 days less). This study demonstrated the safety and feasibility of MIS exenteration in patients with favourable anatomy and tumour characteristics [1]. Moving forward, novel robotic technology such as fluorescence-guided surgery, 3-dimensional modelling and stereotactic navigation will significantly improve surgical dissection and resection margins [93]. Fluorescence-guided surgery was established in several different specialities. Indocyanine green (ICG) can be used to map lymph nodes in various cancers, detect tumour margins and evaluating bowel perfusion at anastomotic sites [94]. Three-dimensional modelling with a virtual reality viewing system is shown to augment the surgical planning process and result in improved patient outcomes. This involves the conversion of CT and MRI images to 3-dimensional virtual reality models helping the surgeon plan the operation [95]. Stereotactic navigation paired with robotics is a novel concept but remains technically challenging. The addition of navigation to robotics will undoubtedly improve surgical precision [96].

#### 2.2.6. Reconstruction

Radical resections incur a greater need for reconstruction. The ability to perform complex soft tissue, vascular and bone reconstruction/stabilization has improved the functional outcomes of patients undergoing pelvic exenteration.

##### Soft Tissue Reconstruction

Many patients will require flap reconstruction after exenterative surgery due to extensive tissue loss. However, some co-existing factors will determine the feasibility of each specific reconstruction. Previous chemoradiotherapy, increased pelvic dead space, poor tissue vascular supply, accumulation of fluid and bacterial contamination all play a role in the development of flap complications, which occur in 25–60% of reconstructions [97]. The following soft tissue reconstruction methods can be considered, based on certain regions [98,99]:

- Abdominal: Vertical or oblique rectus abdominus myocutaneous (VRAM/ORAM).
- Gluteal: Myocutaneous or fascio-cutaneous VY-plasty, inferior gluteal artery perforator (IGAP) flap.
- Upper thigh: Anterolateral thigh +/− vastus lateralis flap, bilateral pedicled gracilis flap.
- Gluteal fold/perineal: Internal pudendal artery perforator or perineal turnover perforator flap.

Vaginal defects resulting from radical oncologic resection are challenging to reconstruct. These defects may range from simple mucosal defects to full circumferential loss due to posterior vaginal wall resection. Anatomy and function can be restored using a rectus abdominis myofascial flap, deep inferior epigastric perforator (DIEP) flap, bilateral gracilis flats or gluteus maximus special flaps [25].

Empty pelvis syndrome is a major contributor to morbidity following pelvic exenteration as dead space allows for the accumulation of fluid and small bowel migration (obstruction) into the pelvis. To alleviate the risk of these complications, the dead space must be “filled” using either synthetic mesh or tissue. Reconstructive methods include myocutaneous flap reconstruction, omental flaps and mesh reconstruction. There is currently insufficient evidence in the literature to support the use of one reconstructive method over another [100].

##### Bony Reconstruction

En bloc sacrectomy is performed in cases where tumours infiltrate the presacral fascia and may require further reconstruction [17]. Sacrectomy results in a large cavity which can result in infection as well as neurological or sexual deficits. Reconstruction aims to restore the pelvic ring and spinopelvic junction [18]. While several fixation methods exist, such as spinopelvic fixation (SPF), posterior pelvic ring fixation (PPRF) and anterior spinal column fixation (ASCF), there is a lack of evidence to suggest the superiority of one method over another [97].

##### Vascular Reconstruction

The involvement of aortoiliac vessels, the sciatic nerve or its associated roots substantially increases the difficulty of achieving an R0 resection in advanced pelvic malignancies [17]. To achieve a clear lateral margin, iliac vessels may be resected en bloc and subsequently reconstructed with an autologous or synthetic graft [17]. A pre-emptive femoral–femoral arterial and venous crossover graft reconstruction method has also been studied and demonstrated a decreased risk of graft infection secondary to avoidance of contamination with gastrointestinal or genitourinary organisms [101,102].

#### 2.2.7. Palliative Surgery

Palliative procedures, such as a defunctioning stoma or urinary diversion, can be performed in patients who are suffering from disabling symptoms where an R0 resection is unlikely [49]. The decision to undergo major palliative surgery in the setting of an advanced/recurrent rectal must be considered on a case-by-case basis [103]. Locally advanced disease can have a profound impact on a patient’s QoL. Relentless growth can cause intractable symptoms, including pain, bleeding, fistulisation to bladder/abdominal wall/bone and/or intestinal obstruction [104]. Increasingly, palliative exenteration is being considered. This remains a controversial topic but should be discussed with patients so that they understand all risks and benefits [49]. Clear counselling, with the involvement of the multidisciplinary team, is vital for establishing treatment goals and expectations [105]. Quyn et al. observed a 62% response rate to their QoL questionnaire using the Functional Assessment of Cancer Therapy—Colorectal (FACT-C). The average FACT-C score returned to pre-operative quality of life two months post-operatively and quality of life continued to improve slowly over the following twelve months [3].

A recent meta-analysis performed by the PelvEx Collaborative demonstrated symptom relief in ‘select patients’ undergoing palliative exenteration. Symptom relief was reported in a median of 79% of patients, although the magnitude of the effect was poorly measured. Though available data are limited (23 studies comprising 509 patients), the results suggest that there may be some improvement in symptom control in selective patients. However, palliative exenteration is an extremely morbid procedure with insufficient evidence of sustained quality of life [49]. As the median overall survival was only 14 months in this cohort of patients, it is essential to consider safer procedures such as stoma formation, bypass, nephrostomy, radiotherapy and multimodal analgesia before resection.

### 2.3. Post-Operative Strategies

Enhanced recovery after surgery (ERAS) protocols are well-established and have demonstrated improvements in morbidity rates, length of stay and quality of life [106]. There is emerging evidence to suggest the feasibility of ERAS in complex cytoreductive surgery with an improvement in early clinical outcomes [107,108,109]. The PelvEx Collaborative has offered guidance on the perioperative management of patients undergoing exenterative surgery and acknowledges the need for individualised tailored post-operative treatment plans [40].

**Figure 1:**
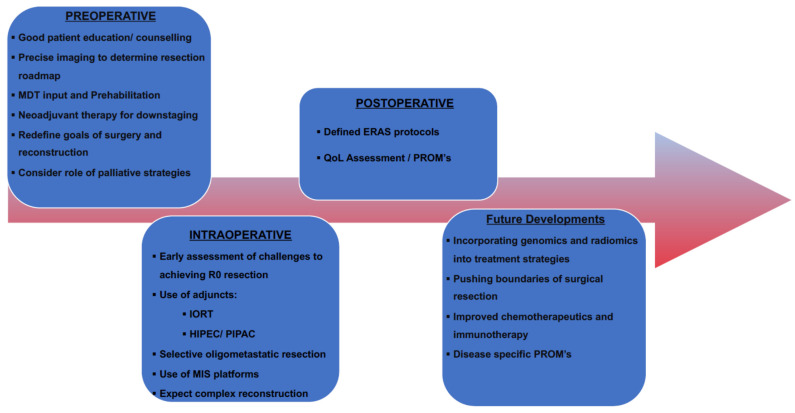
Maximizing success in pelvic exenterative surgery.

Harji et al. enrolled 145 patients into a dedicated pelvic exenteration ERAS programme to assess its feasibility and short-term impact on this cohort of patients. They demonstrated an overall compliance rate of 70%. Patients with higher compliance to the program tended to have a shorter hospital length of stay, reduced rate and severity of post-operative morbidity, as well as fewer readmissions. ERAS appears feasible and efficacious in patients undergoing pelvic exenteration, displaying a high compliance and improved clinical outcomes [110]. See Figure 1 for the highlights of contemporary management strategies.

## 3. Future Developments

The management of advanced colorectal malignancies continues to evolve rapidly, with the introduction of new technologies, pushing the boundaries of surgical resection and involvement in clinical trials and collaborative research. Increasingly, the focus of treatment is balanced between cure and quality of life [111]. The IMPACT initiative highlighted the importance of patient involvement in the decision-making process, incorporating functional and quality outcomes as ‘key’ measures of oncological success [112]. This focus on patient-centred care, combined with burgeoning diagnostic and technological advancements, will continue to shape the approach to colorectal cancer as we move into the era of “personalized medicine”.

### 3.1. Radiomics

Radiomics and radiogenomics are being investigated as a novel way to analyse images and to increase the precision of diagnostics [113]. The incorporation of artificial intelligence with biomarkers will allow clinicians to predict treatment response and may help to personalize care [113,114]. Radiomics can highlight tumour properties throughout serial imaging, and in sufficiently large datasets can uncover previously unknown markers or patterns of disease progression and/or response to chemoradiation [114].

The radiomics analysis of contrast-enhanced CT images has already demonstrated improved accuracy for nodal assessment in advanced rectal cancer [115]. Similarly, a pre-treatment MRI-based machine learning model was developed which can accurately predict treatment response in patients with LARC [116]. These novel technologies may allow for a tailored approach to the treatment of advanced malignancies and help select patients with previously unrecognized adverse features that would benefit from more conservative treatment modalities.

Radiogenomics is the extension of radiomics by its combination with molecular analysis in the form of genomic and transcriptomic data. Genetic analysis remains expensive, invasive, and time-consuming. Radiogenomics may play a vital role in providing imaging surrogates that correlate with genetic expression, thereby providing an alternative to genetic testing [117]. Of course, larger prospective studies with standardization are needed to validate this area of research.

### 3.2. Genomics

The adenoma-carcinoma sequence was first described in 1990 by Vogelstein and Fearon and provided the foundation for our understanding of colorectal cancer as a disease consisting of complex genomic changes [118]. Comparative genomic hybridization (CGH) arrays, single-nucleotide polymorphism (SNP) arrays and novel next-generation sequencing (NGS) approaches have provided us with insights into the complex colorectal cancer genome [119]. Subsequent research has refined and expanded our knowledge of this area, allowing us to incorporate genomics into our treatment choices. The impact of the 100,000 genome project and the integration of genomic and translational medicine into cancer care pathways has provided a unique opportunity for tailoring and personalizing oncological treatment [120]. Research suggests that, in the future, we will be able to predict responses to chemotherapeutics, which will undoubtedly guide decision making, particularly in borderline cases [121]. While the effect of RAS and BRAF mutations is well-established in current clinical practice, new genomic markers are showing promising results in clinical trials [118].

BRAF V600E is the most common potentially targetable mutation in metastatic colorectal cancer; however, RAF inhibitors have limited efficacy as single agents in treating patients with this alteration [118]. The BEACON trial is currently comparing doublet or triplet targeted therapy with standard therapy in patients with BRAF V600E metastatic colorectal cancer [122]. Preliminary results are promising, with a 48% overall response rate in patients receiving triplet therapy consisting of encorafenib (RAF inhibitor), binimetinib (MEK inhibitor) and cetuximab [122].

HER2 amplification occurs in 2–6% of metastatic colorectal cancers and is associated with a poor response to EGFR antibody treatment [118]. While anti-HER2 drugs, such as trastuzumab as monotherapy, have not demonstrated tumour regression in clinical trials, a combination therapy with an EGFR inhibitor achieved tumour shrinkage [123]. The phase 2 HERACLES trial reported a 30% response rate in patients with HER2-positive metastatic colorectal cancer receiving this combination therapy [124]. Cohort B of the HERACLES trial is ongoing and aims to compare the efficacy of the antibody-drug conjugate TDM-1 monotherapy with combination therapy of TDM-1 and pertuzumab (anti-HER2) in the second line setting [118].

Our understanding of tumour biology and molecular subtypes is constantly expanding, thanks to advancements in microarray and NGS technologies which allow for the identification of new cancer genes and pathways. While translational genomic studies have already provided clinically relevant biomarkers for predicting prognosis and therapy response, future research will identify new drug targets and reveal novel therapeutic opportunities. Innovations in current applications, coupled with novel emerging technologies, will lead to further advancements in translational cancer genomics which will hopefully contribute to improved patient outcomes in the future [119].

### 3.3. Pushing the Surgical Boundaries

Over the last two decades, experienced exenterative surgeons have redefined what constitutes resectable disease. The development of regional/national specialized units has allowed funding and structural/service supports to enable these centres to establish specialist pelvic oncology units. As a result, extended bony and/or neurovascular resections are pursued more frequently, with an acceptable morbidity reported [18,65,102].

‘Higher and wider’ resections at the periphery of the pelvis are now commonplace in pelvic exenteration [13]. Certain centres are performing en bloc sciatic nerve and/or lumbosacral trunk excision for tumours that extend laterally into the piriformis. High sacrectomy (above the junction of S2/S3) can be performed without compromising margins, and functional outcomes are acceptable [125]. Alternative techniques such as anterior cortical sacrectomy and abdominolithotomy sacrectomy have become more standardized.

Improved reconstructive techniques have facilitated these more radical resections [21,65,98,99]. However, the repair of large bony defects remains a challenge [126]. The current methods of reconstruction of these defects include autologous iliac grafting, autologous vascularized fibula transplantation, Masquelet’s induced membrane or massive allografts. Autologous grafts account for approximately 50% of cases [126,127]. 3D bioprinting is a state-of-the-art technology used to build constructs from a single-cell type using a layer-by-layer deposition of a specific bioink [127]. Bioprinting uses cell-laden hydrogens to print structures following a period of maturation which can be developed into a variety of complex tissues [126]. Its use in bone reconstruction is still evolving and the clinical application of this technology remains in its infancy. Despite this, bio-printed bone has been successfully implanted in pre-clinical models and other 3D-printed materials have been successfully transplanted into human subjects [128]. This ground-breaking technology will allow us to develop tailored bone grafts that incorporate real cells, growth factors and vasculature, which may revolutionize the way we reconstruct bony defects in the future [126].

Anatomical reconstruction of the sacrum using 3-dimensional printing technology has been sparsely reported in the literature [129,130]. Kim et al. successfully reconstructed the sacrum with a 3D-printed implant in a patient who had undergone hemispherectomy for sacral osteosarcoma. One-year follow up revealed excellent bony union without complication, demonstrating the feasibility of this novel method [129]. Similarly, Chatain et al. described a case of custom 3D-printed sacral implant for revision of failing sacrectomy in a patient who previously underwent en bloc sacrectomy and standard spinopelvic reconstruction for sacral chordoma [130]. In this case, the prosthesis was fashioned from titanium alloy using a 3D-printing technique, tailored to the patient using a CT 3-dimensional reconstruction model. The surgical implantation of the device proved challenging but long-term outcomes were satisfactory [130].

Current options for tissue reconstruction rely heavily on autologous donor tissue to repair defects. 3D bioprinting offers the potential to avoid autologous grafts and the complications associated with them [131]. Its use has now been reported in a variety of surgical disciplines, including plastics, breast, orthopaedic, craniomaxillofacial and head/neck oncology [132]. While significant advancements are being made in the production of simple, single tissue types, composite tissue engineering consisting of multilaminar constructs adds an additional layer of complexity [133]. Ultimately, 3D bioprinting has the potential to produce patient-specific body components, including organs and limbs, which will undoubtedly revolutionize surgery [131].

### 3.4. Histopathology

Magnetic resonance-guided histopathology has been shown to increase the accuracy of staging in LARC [134]. The technique is performed by carefully matching multilevel histologic sections, using previous MR images as guidance, to examine for evidence of residual tumour, while paying particular attention to areas with MRI signals consistent with fibrosis or mucin. Another advancement in the field of histopathology is the use of whole-slide imaging, which produces digital histologic images from glass slides [135]. This technology will allow for the precise evaluation of tumour dimensions, stage and margins, and has the potential to improve both diagnostic accuracy and workflow efficiency in the future.

Biobanking is the process of collecting and storing various human specimens for the purpose of clinical research and provides a platform for the development of translational and personalised “precision” medicine [136]. Translational research with specimens from tissue biobanks enables the discovery of molecular biomarkers that have the potential to guide therapy and individualize treatment [137,138]. Many research programmes have benefited from biobank specimens, including the development of trastuzumab [139]. More recently, biobanks played a crucial role in the creation of The Cancer Genome Atlas (TCGA), a comprehensive catalogue of cancer genomic profiles [140]. This atlas has allowed for the discovery of molecular aberrations at DNA, RNA, protein and epigenetic levels, providing a detailed analysis of commonalities and differences across tumour lineages.

Biobanks provide researchers with human specimens and associated clinical data, which allow for large cohorts of over 30 specimens to be analysed using large-scale genome sequencing. This facilitates the discovery of novel molecular alterations as well as the classification of tumour subtypes according to distinct genomic alterations, providing a personalised, precision medicine approach in cancer care [141].

### 3.5. Quality of Life/PROMs

Aggressive multi-visceral resections are performed more often to manage patients with advanced pelvic cancers [111]. Currently, surgery remains the only long-term curative option in the majority of cases [142]. Despite the radicality of pelvic exenteration, previous studies have demonstrated an acceptable survival rate with reasonable quality of life outcomes [143]. It appears that quality of life scores rapidly deteriorate in the immediate post-operative period; however, they begin to rise slowly again from three months post-operation [144]. The ultimate goal of therapy is to balance patient quality of life with survival and complication rates, and this should be an integral component of the patient counselling process.

Patient counselling with shared decision-making is crucial in the consideration and planning of extensive pelvic surgery. A growing volume of research has shown that when patients are actively involved in decision-making and prehabilitation (nutritional, physiotherapy/conditioning and/or psychological input) that they experience better outcomes [144,145]. The routine use of patient-reported outcome measures (PROMs) will inform us as to how surgery impacts a patient’s lifestyle and quality of life [146,147]. Standardized questionnaires that collect data on patients post-operatively, particularly regarding symptoms, health-related quality of life and functional status are vital [148]. While there is ample support for the use of PROMs in the literature, there has been limited uptake amongst surgeons [149,150]. PROMs not only inform practitioners of the nature, frequency and impact of adverse events following treatment but may also be used to identify and treat these effects on an individual level in the post-operative period. The PelvEx Collaborative has supported the development of a specific PROM QoL tool via the European Organisation for Research and Treatment of Cancer network. Survivorship is at the forefront of this project, in the hope that it will give greater insight into the most important factors affecting patients and strategies. The morbidity of pelvic exenterative surgery may extend long into the months after discharge from hospital, and it is imperative that we have the necessary supports in place to manage these complications [151]. Post-operative specialist multi-disciplinary care is essential to assist patients with pain, wound and stoma management as well as for psychological support.

## 4. Conclusions

The role of radical surgery in the setting of locally advanced and recurrent rectal cancer has evolved substantially. Novel strategies including TNT, cytoreductive and/or bony/vascular resection and enhanced reconstructive techniques have enabled surgeons to pursue what was once considered a terminal disease. Advancements in surgical technology, in particular the incorporation of artificial intelligence and three-dimensional bioprinting, will undoubtedly enhance our ability to move the limits of what is reasonable and possible to resect.

## Appendix A

PelvEx Collaborative: Kelly M.E., O’Sullivan N.J., Fahy M.R., Aalbers A.G.J., Abdul Aziz N., Abecasis N., Abraham-Nordling M., Abu Saadeh F., Akiyoshi T., Alberda W., Albert M., Andric M., Angeles M.A., Angenete E., Antoniou A., Auer R., Austin K.K., Aytac E., Aziz O., Bacalbasa N., Baker R.P., Bali M., Baransi S., Baseckas G., Bebington B., Bedford M., Bednarski B.K., Beets G.L., Berg P.L., Bergzoll C., Beynon J., Biondo S., Boyle K., Bordeianou L., Brecelj E., Bremers A.B., Brunner M., Buchwald P., Bui A., Burgess A., Burger J.W.A., Burling D., Burns E., Campain N., Carvalhal S., Castro L., Caycedo-Marulanda A., Ceelen W., Chan K.K.L., Chang G.J., Chang M., Chew M.H., Chok A.Y., Chong P., Clouston H., Codd M., Collins D., Colquhoun A.J., Constantinides J., Corr A., Coscia M., Cosimelli M., Cotsoglou C., Coyne P.E., Croner R.S., Damjanovich L., Daniels I.R., Davies M., Delaney C.P., de Wilt J.H.W., Denost Q., Deutsch C., Dietz D., Domingo S., Dozois E.J., Drozdov E., Duff M., Eglinton T., Enriquez-Navascues J.M., Espín-Basany E., Evans M.D., Eyjólfsdóttir B., Fearnhead N.S., Ferron G., Fichtner-Feigl S., Flatmark K., Fleming F.J., Flor B., Folkesson J., Frizelle F.A., Funder J., Gallego M.A., Gargiulo M., García-Granero E., García-Sabrido J.L., Gargiulo M., Gava V.G., Gentilini L., George M.L., George V., Georgiou P., Ghosh A., Ghouti L., Gil-Moreno A., Giner F., Ginther D.N., Glyn T., Glynn R., Golda T., Griffiths B., Harris D.A., Hanchanale V., Harji D.P., Harris C., Helewa R.M., Hellawell G., Heriot A.G., Hochman D., Hohenberger W., Holm T., Hompes R., Hornung B., Hurton S., Hyun E., Ito M., Iversen L.H., Jenkins J.T., Jourand K., Kaffenberger S., Kandaswamy G.V., Kapur S., Kanemitsu Y., Kazi M., Kelley S.R., Keller D.S., Ketelaers S.H.J., Khan M.S., Kiran R.P., Kim H., Kim H.J., Koh C.E., Kok N.F.M., Kokelaar R., Kontovounisios C., Kose F., Koutra M., Kristensen H.Ø., Kroon H.M., Kumar S., Kusters M., Lago V., Lampe B., Lakkis Z., Larach J.T., Larkin J.O., Larsen S.G., Larson D.W., Law W.L., Lee P.J., Limbert M., Loria A., Lydrup ML., Lyons A., Lynch A.C., Maciel J., Manfredelli S., Mann C., Mantyh C., Mathis K.L., Marques C.F.S., Martinez A., Martling A., Mehigan B.J., Meijerink W.J.H.J., Merchea A., Merkel S., Mehta A.M., Mikalauskas S., McArthur D.R., McCormick J.J., McCormick P., McDermott F.D., McGrath J.S., Malde S., Mirnezami A., Monson J.R.T., Navarro A.S., Neeff H., Negoi I., Neto J.W.M., Ng J.L., Nguyen B., Nielsen M.B., Nieuwenhuijzen G.A.P., Nilsson P.J., Nordkamp S., Nugent T., Oliver A., O’Dwyer S.T., Paarnio K., Palmer G., Pappou E., Park J., Patsouras D., Peacock O., Pellino G., Peterson A.C., Pfeffer F., Pinson J., Poggioli G., Proud D., Quinn M., Quyn A., Rajendran N., Radwan R.W., Rajendran N., Rao C., Rasheed S., Rausa E., Regenbogen S.E., Reims H.M., Renehan A., Rintala J., Rocha R., Rochester M., Rohila J., Rothbarth J., Rottoli M., Roxburgh C., Rutten H.J.T., Safar B., Sagar P.M., Sahai A., Saklani A., Sammour T., Sayyed R., Schizas A.M.P., Schwarzkopf E., Scripcariu D., Scripcariu V., Selvasekar C., Shaikh I., Simpson A., Skeie-Jensen T., Smart N.J., Smart P., Smith J.J., Solbakken A.M., Solomon M.J., Sørensen M.M., Sorrentino L., Steele S.R., Steffens D., Stitzenberg K., Stocchi L., Stylianides N.A., Swartling T., Spasojevic M., Sumrien H., Sutton P.A., Swartking T., Takala H., Tan E.J., Taylor C., Taylor D., Tekin A., Tekkis P.P., Teras J., Thaysen H.V., Thurairaja R., Thorgersen E.B., Tiernan J., Toh E.L., Tolenaar J., Tsarkov P., Tsukada Y., Tsukamoto S., Tuech J.J., Turner W.H., Tuynman J.B., Valente M., van Ramshorst G.H., van Rees J., van Zoggel D., Vasquez-Jimenez W., Vather R., Verhoef C., Vierimaa M., Vizzielli G., Voogt E.L.K., Uehara K., Urrejola G., Wakeman C., Warrier S.K., Wasmuth H.H., Waters P.S., Weber K., Weiser M.R., Wheeler J.M.D., Wild J., Williams A., Wilson M., Wolthuis A., Yano H., Yip B., Yoo R.N., Zappa M.A., Winter D.C.
